# Characteristics and Outcomes of 1500 Lung Transplantations in the Leuven Lung Transplant Program: Turning Past Lessons Into Tomorrow’s Foundations

**DOI:** 10.3389/ti.2025.15495

**Published:** 2025-11-12

**Authors:** Andrea Zajacova, Lieven J. Dupont, Paul De Leyn, Laurens J. Ceulemans, Robin Vos, Leuven Lung Transplant Group

**Affiliations:** 1 Department of Respiratory Diseases, University Hospitals Leuven, Leuven, Belgium; 2 Department CHROMETA, Laboratory of Respiratory Diseases and Thoracic Surgery (BREATHE), KU Leuven, Leuven, Belgium; 3 Department of Thoracic Surgery, University Hospitals Leuven, Leuven, Belgium

**Keywords:** lung transplantation, outcome, graft survival, evolution over time, future perspectives

## Abstract

Lung transplantation has become an established life-saving treatment for selected patients with end-stage pulmonary disease. In December 2024, our center reached the milestone of 1,500 lung transplants, providing an opportunity to evaluate long-term trends, outcomes, and challenges. We analyzed donor and recipient demographics, procedural evolution, and graft survival. Contemporary guidelines and consensus recommendations were also reviewed to contextualize current practice and highlight unmet needs. Median graft survival improved markedly across eras: 3.5 years between 1991 and 2000, 9.9 years between 2001 and 2010, and 11.2 years between 2011 and 2020 (p < 0.0001). Shifts in procedure type, donor selection, and transplant indications mirrored broader developments in the field (all p < 0.0001). Donor and recipient age increased significantly over time, with older recipients experiencing poorer long-term outcomes. Despite these advances, chronic lung allograft dysfunction (CLAD) remains the most important barrier to durable success, with median CLAD-free survival of 6.7 years in the modern era (2010–2024) and a retransplantation rate of 4%. While survival now exceeds a decade in many recipients, extended longevity presents new challenges, including management of comorbidities and optimization of CLAD prevention, treatment, and retransplantation strategies. Continued translational research and evidence-based approaches remain critical to improving long-term results.

## Graphical Abstract

**Figure:**
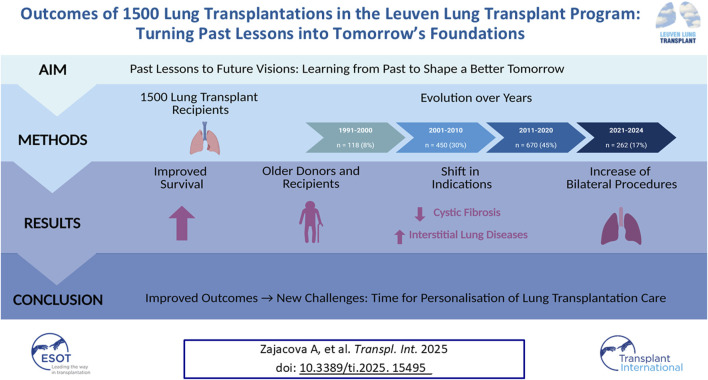

## Introduction

The first human lung transplantation (LuTx), performed by James Hardy in 1963, demonstrated technical feasibility of this procedure, but initial post-transplant outcome was poor [1]. Introduction of cyclosporine A into clinical practice in the early 1980s, combined with advances in surgical techniques, marked the beginning of the modern era of LuTx [2]. Since then, the annual number of LuTx procedures has steadily increased, now estimated to globally exceed over 5,500 transplantations per year, with in total more than 70,000 procedures performed to date [3, 4]. Notably, current registries (i.e., International Society for Heart and Lung Transplantation (ISHLT), Organ Procurement and Transplantation Network (OPTN), Collaborative Transplant Study (CTS), etc.) fail to capture all individual procedures performed world-wide, since reporting is not mandatory in every transplant center, and exact global transplant numbers are therefore unclear – and likely underestimated [3, 4]. Moreover, not all centers provide data on post-transplant outcomes, making actual graft survival—particularly long-term results—often uncertain. This underscores the need for better reporting of transplant centers’ outcomes.

Today, LuTx has become an established treatment option for carefully selected patients with end-stage pulmonary diseases. Advances in medical therapies and management strategies for respiratory conditions such as chronic obstructive pulmonary disease (COPD), interstitial lung diseases (ILD), pulmonary arterial hypertension, and cystic fibrosis (CF)—the main indications for LuTx—have significantly influenced patient selection and referral patterns over the past decades, as well as post-transplant outcomes [3]. These developments also shaped ISHLT guidelines and referral recommendations over time [5, 6]. In parallel, improvements in donor management and optimized surgical techniques, along with introduction of novel, innovative technological approaches such as extracorporeal life support bridging, controlled temperature organ preservation, and *ex vivo* lung perfusion, are nowadays transforming LuTx from an urgent, unplanned intervention into a more predictable, even scheduled, surgical procedure [7–9]. While ISHLT-endorsed recommendations have provided valuable guidance for pre-, peri- and post-transplant patient care ([3, 10–26]; Supplementary Table S1), immune-mediated complications remain a major challenge for improving long-term outcomes. Chronic lung allograft dysfunction (CLAD) continues to limit long-term survival, with current median graft survival reported at just 6.3 years, according to the ISHLT Registry [27].

At our center, the 1,500th LuTx was performed in December 2024, an achievement that prompted a comprehensive analysis of our cohort’s donor and recipient characteristics, surgical approaches, and long-term outcomes, to evaluate trends and progress over time, and to identify current challenges and conceptional unmet needs to further improve future long-term patient care.

## Materials and Methods

### Study Population

This retrospective, single-center study analyzed all 1,500 consecutive LuTx procedures performed at University Hospitals Leuven between July 1991 and December 12, 2024. Data collected included donor demographics (age and donation type) and recipient characteristics (age at LuTx, sex, transplant indication, date of transplant, procedure type, time to CLAD, CLAD phenotype, and graft survival), with follow-up censored on 31 December 2024. Patients were categorized by transplantation era (1991–2000, 2001–2010, 2011–2020, and 2021–2024) and by procedure type: unilateral LuTx (single LuTx), bilateral LuTx (sequential single LuTx), or combined LuTx (LuTx combined with heart, liver, and/or kidney transplantation). Transplant indications were grouped into four categories: obstructive, restrictive, vascular, and CF) (Supplementary Table S2) for outcome analyses. For patients transplanted since 2010, CLAD phenotyping was performed according to the 2019 ISHLT consensus [12], as earlier data were insufficient for detailed classification. Institutional Ethical Review Board approval was waived for this retrospective observational study (S51577/S63978).

### Statistical Analysis

Statistical analyses and visualizations were performed using GraphPad Prism 10.4.0 (San Diego, CA, United States). Categorical variables were analyzed with Fisher’s exact test and Chi-square test, while continuous variables were assessed using the Kruskal-Wallis test. Survival outcomes were evaluated using log-rank tests and illustrated with Kaplan-Meier curves.

## Results

### Patient Cohort and Graft Survival

The number of transplant procedures increased steadily over time: 8% of our cohort underwent transplantation between 1991 and 2000, 30% between 2001 and 2010, 45% between 2011 and 2020, and the remaining 17% of transplantations were performed between 2021 and 2024. Recipient and donor demographics are summarized in Supplementary Tables S3, S4. At the censoring date, 746 recipients (50%) were alive and followed up in our center.

**FIGURE 1:**
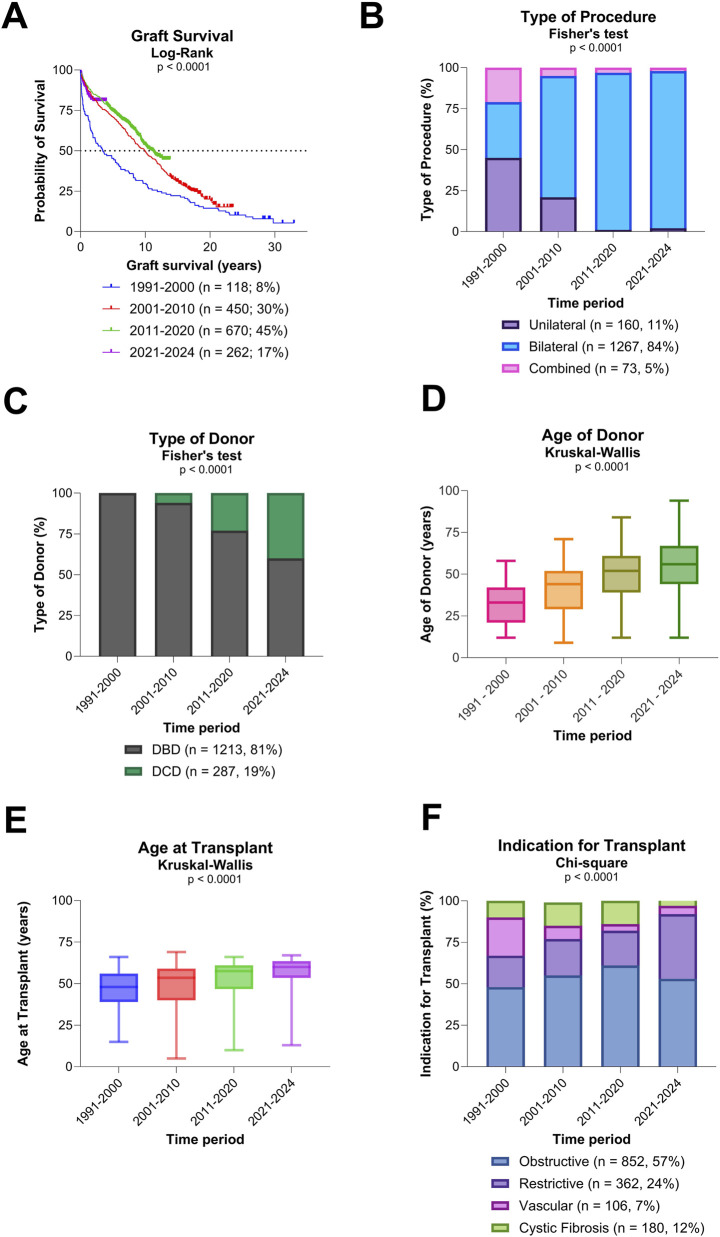
Overall graft survival, type of procedure, and donor/recipient characteristics **(A)** Evolution of overall graft survival over time. **(B)** Evolution of type of procedure over time. **(C)** Evolution of type of donors over time. **(D)** Evolution of age of donors over time. **(E)** Evolution of recipient age at transplantation over time. **(F)** Evolution of indication for transplantation over time.

Overall graft survival among all 1,500 patients was 88% at 1 year, 78% at 3 years, 70% at 5 years, 50% at 10 years, 33% at 15 years, and 22% at 20 years. Graft survival improved significantly across eras, with median survival increasing from 3.5 years (1991–2000; 95% CI 1.9–6) to 9.9 years (2001–2010; 95% CI 8.9–11.4), and 11.2 years (2011–2020; 95% CI 9.8–NA) (p < 0.0001; Figure 1A). Conditional 1-year graft survival also improved, from 7.8 years (1991–2000; 95% CI 5.5–10.6) to 11.5 years (2001–2010; 95% CI 10.4–12.6), and 12.6 years (2011–2020; 95% CI 11.44–NA) (p = 0.003).

### Donor and Recipient Characteristics

A significant shift towards bilateral LuTx was observed, along with a significant increased use of donors after circulatory death (DCD) (both p < 0.0001; Figures 1B,C). Additionally, both donor and recipient ages rose significantly over time (both p < 0.0001; Figures 1D,E). There was a notable change in the indications for LuTx: the proportion of patients transplanted for CF declined, while those with ILD increased (p < 0.0001; Figure 1F).

**FIGURE 2:**
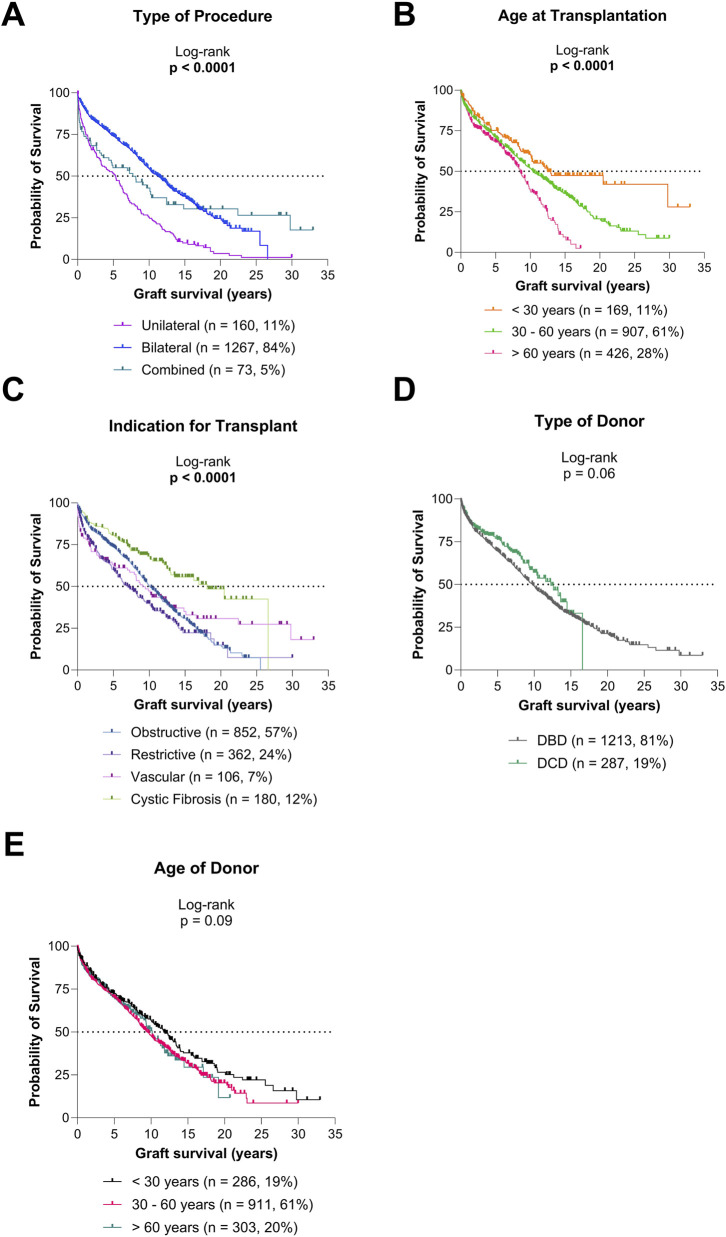
Graft survival according to type of procedure and donor/recipient characteristics **(A)** Graft survival for diverse types of procedures. **(B)** Graft survival for diverse recipient age groups. **(C)** Graft survival for diverse indications for transplantation. **(D)** Graft survival based on type of donor. **(E)** Graft survival based on age of donor.

Post-transplant survival varied significantly by type of LuTx, recipient age, and indication for transplantation (all p < 0.0001; Figures 2A–C). Trends toward different survival outcomes were observed by donor type (p = 0.06; Figure 2D) and donor age (p = 0.09; Figure 2E), though these did not reach statistical significance. Outcomes for each indication group (obstructive, restrictive, vascular, and CF) and single LuTx alone are shown in Supplementary Figures S1–S5.

### CLAD and Retransplantation

**FIGURE 3:**
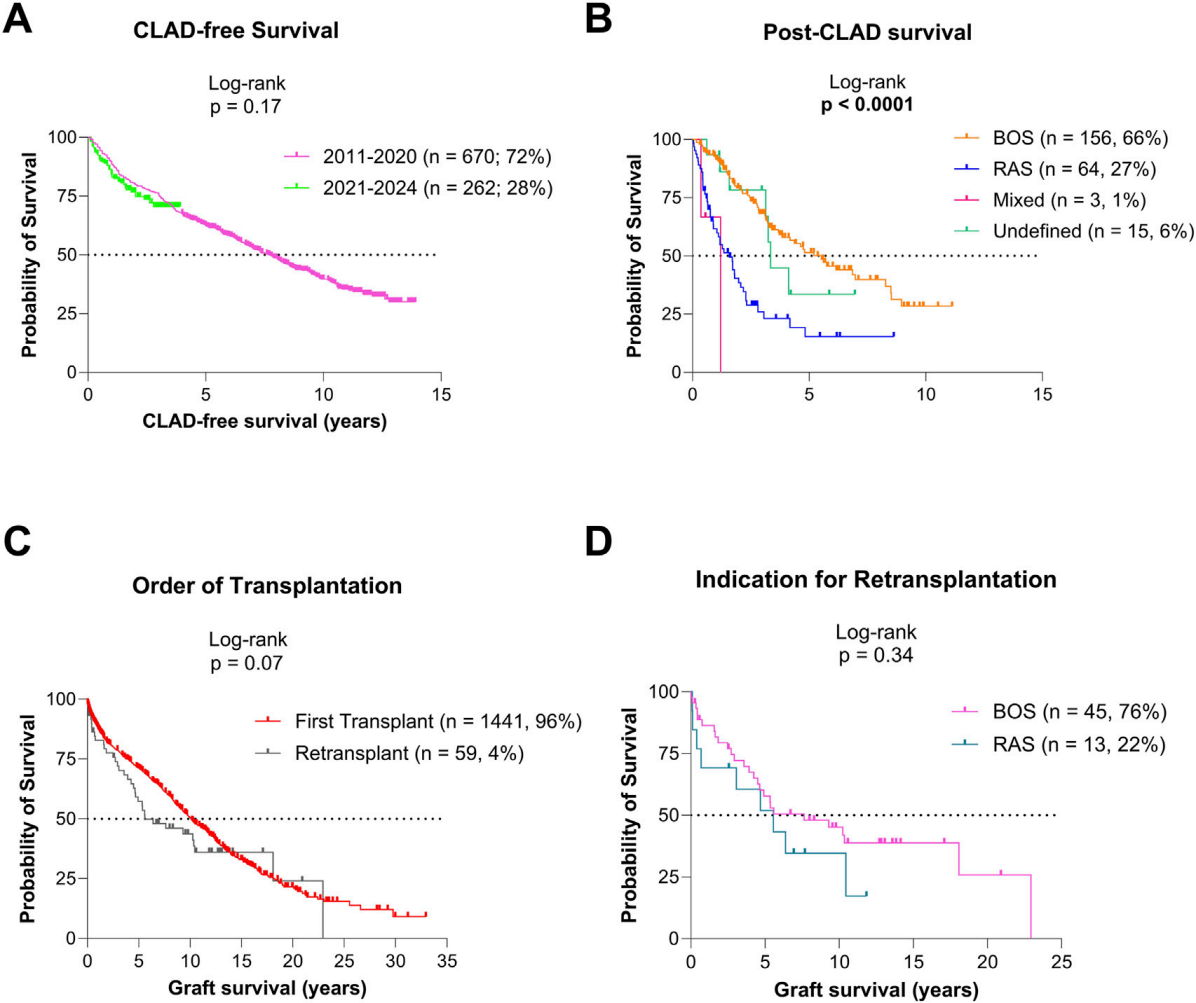
Graft survival based on CLAD status **(A)** CLAD-free survival for the patients transplanted since 2011. **(B)** Post-CLAD survival for patients transplanted since 2011. **(C)** Graft survival for patients after primary transplantation and retransplantation. **(D)** Graft survival after retransplantation for BOS and RAS CLAD phenotypes.

For the cohort included in CLAD analysis (2011–2024, n = 989; 66%), median CLAD-free survival was 7.3 years (95% CI 6.6–8.2; Figure 3A). CLAD-free survival was comparable in patients transplanted in 2011–2020 vs. 2021–2024 (p = 0.17). Among the 238 patients (25.5%) who developed CLAD, 66% had bronchiolitis obliterans syndrome (BOS), 27% restrictive allograft syndrome (RAS), 1% mixed phenotype, and 6% undefined phenotype. Post-CLAD survival differed significantly by phenotype: 5.5 years for BOS (95% CI 3.8–8.5), 1.6 years for RAS (95% CI 1–2.2), 1.2 years for mixed (95% CI 0.37-NA), and 3.4 years for undefined phenotypes (95% CI 3.13-NA; p < 0.0001; Figure 3B).

In total, 59 lung retransplantations (reLuTx; 4%) were performed: 45 (76%) for BOS, 13 (22%) for RAS, and 1 (2%) for early postoperative pulmonary venous occlusion. Graft survival after reLuTx tended to be lower than after primary LuTx (p = 0.07), but survival between BOS and RAS indications was similar (p = 0.34; Figures 3C,D).

## Discussion

Over the course of 1,500 lung transplants in Leuven, graft survival has significantly increased from 3.5 to 11.2 years, notwithstanding major changes in procedure types, and donor and recipient profiles -trends that align with ISHLT Registry data and findings from other large cohorts [3, 28]. These improvements cannot be attributed to a single factor, but rather reflect the cumulative effect of multiple, coordinated advances in surgical techniques, perioperative management, immunosuppressive therapies, infection prophylaxis, and long-term follow-up care of recipients. These changes, combined with general improvements in medical management, complicate direct comparisons of patient cohorts across different time periods and centers. This emphasizes the importance of contemporary, near real-time outcome assessments over reliance on pooled historical registry data.

Unilateral LuTx, once predominant between 1991 and 2000, has declined steadily since 2011. This change likely reflects advances in donor management, expanding donor criteria with increase in DCD donations and broader use of older donors [29], improved graft preservation techniques, and refined surgical practices, which altogether have expanded the donor pool while reducing surgical risks and complications of bilateral transplantation, which is now the preferred procedure in most centers. Given that unilateral LuTx confers inferior long-term graft survival compared to bilateral LuTx, this may raise ethical concerns when prioritizing unilateral over bilateral LuTx, despite donor shortages and patient factors (e.g., older age) which could favor its use. Unfortunately, evidence-based guidelines for the selection of appropriate candidates for ‘split’ (dividing two suitable donor lungs between recipients) or ‘isolated’ single LuTx (where one donor lung is transplanted, and the other is declined) are still lacking [30]. In general, most centers nowadays mainly reserve unilateral LuTx for selected cases with either ILD or emphysema - in both of which conditions significant challenges may arise during long-term follow-up (i.e., infections or malignancy in the native lung, progressive fibrosis in ILD, hyperinflation in emphysema), which may compromise patient outcome.

Due to the aging LuTx population, other challenges also arise. The proportion of COPD recipients over 60 years increased from ∼25% (1992–2000) to over 50% (2010–2018) per ISHLT data [31]. This is in line with evolving ISHLT candidate selection recommendations: in 2014, age >65 years was a relative contraindication, while in 2021, age 65–70 years became a risk factor only [6, 7]. However, older recipients face worse long-term outcomes [3], as also seen in our cohort, which again may raise ethical concerns when listing elderly patients. Our center’s LuTx listing age limit is 65 years (67 for ILD), based on ISHLT registry data consistently demonstrating an increased risk for post-transplant mortality in patients aged ≥60 years [27]. Especially for elderly candidates, thorough comorbidity and frailty assessments, prehabilitation, and enhanced recovery protocols are essential [32–34]. Yet, optimal frailty evaluation and management remain undefined, highlighting the need for guidelines on pretransplant frailty assessment, prehabilitation, and post-transplant physiotherapy protocols along with comorbidity management.

CLAD remains one of the main factors limiting long-term survival after lung transplantation. Despite advances in graft monitoring strategies and improved recognition of CLAD and its clinical phenotypes over the past decade, little progress has been made in the treatment of this devastating complication. Consequently, the development of evidence-based guidelines for CLAD prevention and management, together with sustained translational research to elucidate its underlying mechanisms and clinical trials testing novel therapies, represents a critical and unmet need. Meanwhile, reLuTx—still the only curative option for CLAD—is becoming increasingly common worldwide, yet clear referral and listing criteria remain absent [3, 35]. Importantly, long-term use of maintenance immunosuppressive therapies–the cornerstone of transplant medicine–frequently leads to non-respiratory comorbidities (i.e., cardiovascular and renal disease, diabetes, or malignancy), which may contribute to poorer outcomes in older patients following primary LuTx, and especially after reLuTx, where the cumulative burden of immunosuppressive therapy increases substantially over time [36, 37]. Notably, reLuTx may pose significant technical challenges, especially in restrictive CLAD/RAS (i.e., pleural adhesions), which may contribute to the worse outcomes in reLuTx compared to primary LuTx [36, 37]. While previous studies reported significantly shorter post-transplant survival in reLuTx for restrictive CLAD/RAS compared to obstructive CLAD/BOS, our cohort did not fully mirror these findings [38, 39]. More strict candidate selection following recent ISHLT recommendations [6] and increased surgical experience with performing reLuTx over time [35] may have contributed to this result [6, 35]. Interestingly, the incidence of RAS as indication for reLuTx rose sharply over time in our cohort, from 6% (2001–2010) to 29% (2011–2020), and 40% (2021–2024) (Supplementary Table S3), likely reflecting improved recognition of this phenotype since its description in 2011 [40], as well as the worse prognosis associated with this phenotype compared to BOS, which may skew referrals towards reLuTx listing. Importantly, given the overall poor prognosis associated with CLAD, timely reLuTx evaluation in patients with CLAD—particularly at CLAD stage 4 (FEV1 ≤35% of post-transplant baseline)—is critical. Thorough multidisciplinary pre-reLuTx assessment is essential, and despite formal reLuTx criteria are currently missing, listing otherwise eligible patients could be considered when FEV1 and/or DLCO are ≤30% predicted, especially in the presence of pulmonary arterial hypertension or exertional hypoxemia.

While extending long-term survival and enhancing quality of life remain the primary goals of LuTx, appropriate end-of-life care is an essential component of management for all recipients. Early and proactive advance care planning is particularly important for patients who develop respiratory complications such as CLAD or who experience severe non-respiratory comorbidities. Future recommendations should address terminal-stage management, including symptom management, reduction of polypharmacy, and palliative care. Finally, with an ever-growing LuTx population requiring extended and often complex healthcare, also adequate healthcare organization—including logistical coordination, budgetary management, workload control, and physicians’ well-being—requires attention. Adequate staffing to ensure state-of-the-art, life-long patients’ follow-up and to avoid burnout in healthcare practitioners is challenging, yet essential for sustainable high-quality transplant care [41, 42]. Table 1 highlights some of the essential areas for future clinical guidelines, which may be pivotal in further optimizing long-term outcomes. Supplementary Table S5 provides a list of ISHLT-endorsed guidelines currently in development.

**TABLE 1:** Key areas of focus for future clinical guidelines to improve long-term outcomes.

Pretransplant care
Frailty assessment criteria
Prehabilitation strategies
Lung retransplant criteria
Peritransplant care
Donor optimization strategies
Organ preservation strategies
Immunosuppression strategies and management of immunologically sensitized recipients
Management of surgical complications, including bronchial anastomosis problems
Early recovery after surgery protocols and post-transplant revalidation strategies
Posttransplant care
Maintenance immunosuppression regimen strategies
Prevention and treatment of chronic lung allograft dysfunction
Screening and management of comorbidities
Patient reported outcomes
Advanced care planning and end-of-life management
Healthcare practitioners’ involvement
Healthcare organization, including logistics, workload and budgetary management

In conclusion, long-term outcomes post-LuTx are nowadays favorable, with median graft survival exceeding a decade in our center. However, changing donor and recipient profiles, and longer post-transplant survival conveys new challenges, of which the most important remains CLAD. Our findings may also help inform future clinical guidelines by illustrating how evolving donor and recipient characteristics, shifting indications, and changes in transplant types impact outcomes. Ongoing translational research efforts, systematic outcome assessment, and evidence-based strategies are essential to address these challenges and further improve long-term results in LuTx. Recommendations on evidence-based strategies regarding frailty assessment and management, post-transplant comorbidities, CLAD prevention and treatment, reLuTx criteria, and end-of-life care are crucial to advancing current lung transplant care.

## Data Availability

The data analyzed in this study is subject to the following licenses/restrictions: The datasets analyzed during this study are not publicly available because of patient privacy concerns and institutional data protection policies. Requests to access these datasets should be directed to robin.vos@uzleuven.be.

## Group Members of Leuven Lung Transplant Group

Saskia Bos: University Hospitals Leuven, Department of Respiratory Diseases, KU Leuven, Department CHROMETA, BREATHE, Leuven, Belgium; Laurens De Sadeleer: University Hospitals Leuven, Department of Respiratory Diseases, KU Leuven, Department CHROMETA, BREATHE, Leuven, Belgium; Laurent Godinas: University Hospitals Leuven, Department of Respiratory Diseases, KU Leuven, Department CHROMETA, BREATHE, Leuven, Belgium; Dirk E. Van Raemdonck, KU Leuven, Department CHROMETA, BREATHE, Leuven, Belgium, University Hospitals Leuven, Department of Thoracic Surgery; Lieven Depypere, KU Leuven, Department CHROMETA, BREATHE, Leuven, Belgium, University Hospitals Leuven, Department of Thoracic Surgery; Hans Van Veer, KU Leuven, Department CHROMETA, BREATHE, Leuven, Belgium, University Hospitals Leuven, Department of Thoracic Surgery; Yanina Jansen, KU Leuven, Department CHROMETA, BREATHE, Leuven, Belgium, University Hospitals Leuven, Department of Thoracic Surgery; Catherine Ingels, University Hospitals Leuven, Department of Intensive Care; Karen Denaux, University Hospitals Leuven, Organ Donation and Transplant Coordination; Arne P. Neyrinck, University Hospitals Leuven, Department of Anesthesiology; Filip Rega, KU Leuven, Department CHROMETA, BREATHE, Leuven, Belgium, University Hospitals Leuven, Department of Thoracic Surgery; Bart M. Vanaudenaerde, KU Leuven, Department CHROMETA, BREATHE, Leuven, Belgium. All listed group members contributed to the reviewing of this article.
