# Supplementary material for: Characteristics and Outcomes of 1500 Lung Transplantations in the Leuven Lung Transplant Program: Turning Past Lessons Into Tomorrow’s Foundations
Source: Transpl Int. 2025 Nov 12;38:15495. doi: 10.3389/ti.2025.15495 (PMC12648049; doi:10.3389/ti.2025.15495)
Supplement: Supplementary file 1 [file DataSheet2.pdf]

Supplementary Figure 1: Evolution of graft survival, type of procedure and recipient age at lung transplantation across eras for the obstructive indication group (A, B, and C, respectively).

Supplementary Figure 2: Evolution of graft survival, type of procedure and recipient age at lung transplantation across eras for the restrictive indication group (A, B, and C, respectively).

Supplementary Figure 3: Evolution of graft survival, type of procedure and recipient age at lung transplantation across the decades for the vascular indication group (A, B, and C, respectively).

Supplementary Figure 4: Evolution of graft survival, type of procedure and recipient age at lung transplantation across eras for the cystic fibrosis indication group (A, B, and C, respectively).

Supplementary Figure 5: Evolution of graft survival after unilateral LuTx (single lung transplant; SLuTx)
