# Supplementary figures and images for: Characteristics and Outcomes of 1500 Lung Transplantations in the Leuven Lung Transplant Program: Turning Past Lessons Into Tomorrow’s Foundations
Source: Transpl Int. 2025 Nov 12;38:15495. doi: 10.3389/ti.2025.15495 (PMC12648049; doi:10.3389/ti.2025.15495)

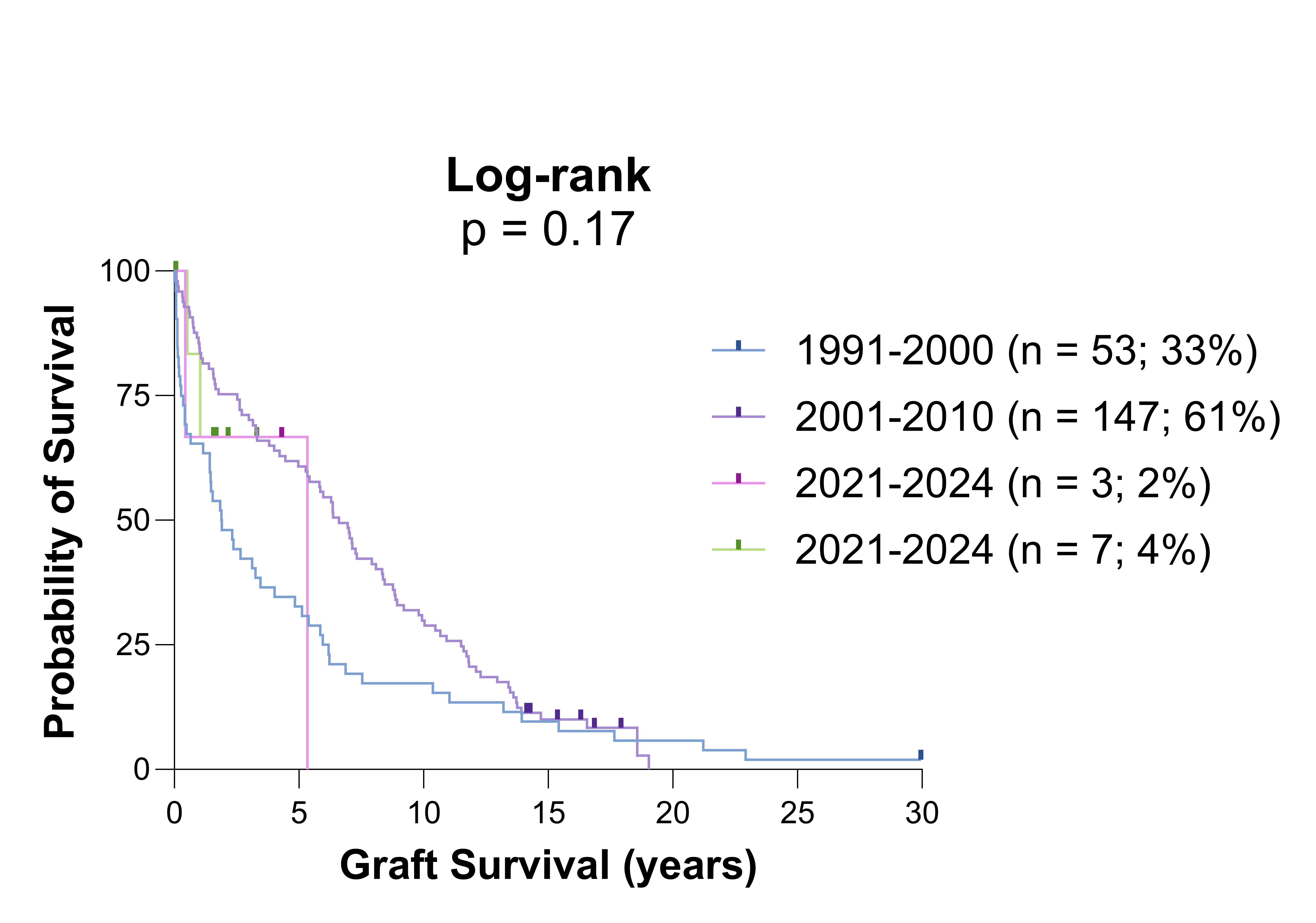

Supplement: Supplementary file 6 [file Image5.jpeg]
