# Supplementary material for: Characteristics and Outcomes of 1500 Lung Transplantations in the Leuven Lung Transplant Program: Turning Past Lessons Into Tomorrow’s Foundations
Source: Transpl Int. 2025 Nov 12;38:15495. doi: 10.3389/ti.2025.15495 (PMC12648049; doi:10.3389/ti.2025.15495)
Supplement: Supplementary file 7 [file DataSheet1.pdf]

Supplementary Table 1: Practical guidelines and consensus statements on pre-, peri-, and post-transplant care of lung transplant recipients, endorsed by the International Society for Heart and Lung Transplantation in reverse chronological order.

	Year	Publication	Reference
**Pretransplant**	2022	International Society for Heart and Lung Transplantation statement on transplant ethics	Holm AM, Fedson S, Courtwright A, et al. International society for heart and lung transplantation statement on transplant ethics. J Heart Lung Transplant. 2022;41(10):1307-1308. doi:10.1016/j.healun.2022.05.012
	2021	Leard LE, Holm AM, Valapour M, et al. Consensus document for the selection of lung transplant candidates: An update from the International Society for Heart and Lung Transplantation	Leard LE, Holm AM, Valapour M, et al. Consensus document for the selection of lung transplant candidates: An update from the International Society for Heart and Lung Transplantation. J Heart Lung Transplant. 2021;40(11):1349-1379. doi:10.1016/j.healun.2021.07.005
	2021	ISHLT consensus document on lung transplantation in patients with connective tissue disease: Part I: Epidemiology, assessment of extrapulmonary conditions, candidate evaluation, selection criteria, and pathology statements	Crespo MM, Lease ED, Sole A, et al. ISHLT consensus document on lung transplantation in patients with connective tissue disease: Part I: Epidemiology, assessment of extrapulmonary conditions, candidate evaluation, selection criteria, and pathology statements. J Heart Lung Transplant. 2021;40(11):1251-1266. doi:10.1016/j.healun.2021.07.014
	2018	The 2018 ISHLT/APM/AST/ICCAC/STSW Recommendations for the Psychosocial Evaluation of Adult Cardiothoracic Transplant Candidates and Candidates for Long-Term Mechanical Circulatory Support	Dew MA, DiMartini AF, Dobbels F, et al. The 2018 ISHLT/APM/AST/ICCAC/STSW recommendations for the psychosocial evaluation of adult cardiothoracic transplant candidates and candidates for long-term mechanical circulatory support. J Heart Lung Transplant. 2018;37(7):803-823. doi:10.1016/j.healun.2018.03.005
	2014	A consensus document for the selection of lung transplant candidates: 2014--an update from the Pulmonary Transplantation Council of the International Society for Heart and Lung Transplantation	Weill D, Benden C, Corris PA, et al. A consensus document for the selection of lung transplant candidates: 2014--an update from the Pulmonary Transplantation Council of the International Society for Heart and Lung Transplantation. J Heart Lung Transplant. 2015;34(1):1-15. doi:10.1016/j.healun.2014.06.014
	2006	International Guidelines for the Selection of Lung Transplant Candidates: 2006 Update	Orens JB, Estenne M, Arcasoy S, et al. International guidelines for the selection of lung transplant candidates: 2006 update--a consensus report from the Pulmonary Scientific Council of the International Society for Heart and Lung Transplantation. J Heart Lung Transplant. 2006;25(7):745-755. doi:10.1016/j.healun.2006.03.011
**Peritransplant**	2024	ISHLT consensus statement on the	Martin AK, Mercier O, Fritz AV, et al. ISHLT consensus

		perioperative use of ECLS in lung transplantation: Part II: Intraoperative considerations	statement on the perioperative use of ECLS in lung transplantation: Part II: Intraoperative considerations. J Heart Lung Transplant. Published online October 9, 2024. doi:10.1016/j.healun.2024.08.027
	2024	ISHLT Consensus Statement on the Perioperative use of ECLS in Lung Transplantation: Part III - Postoperative Considerations	Martin AK, Mercier O, Bottiger B, et al. ISHLT consensus statement on the perioperative use of ECLS in lung transplantation: Part III: Postoperative considerations. J Heart Lung Transplant. Published online April 7, 2025. doi:10.1016/j.healun.2025.03.004
	2022	ISHLT position paper on thoracic organ transplantation in controlled donation after circulatory determination of death (cDCD)	Holm AM, Courtwright A, Olland A, Zuckermann A, Van Raemdonck D. ISHLT position paper on thoracic organ transplantation in controlled donation after circulatory determination of death (cDCD). J Heart Lung Transplant. 2022;41(6):671-677. doi:10.1016/j.healun.2022.03.005
	2021	International consensus recommendations for anesthetic and intensive care management of lung transplantation. An EACTAIC, SCA, ISHLT, ESOT, ESTS, and AST approved document	Marczin N, de Waal EEC, Hopkins PMA, et al. International consensus recommendations for anesthetic and intensive care management of lung transplantation. An EACTAIC, SCA, ISHLT, ESOT, ESTS, and AST approved document. J Heart Lung Transplant. 2021;40(11):1327-1348. doi:10.1016/j.healun.2021.07.012
	2021	ISHLT consensus document on lung transplantation in patients with connective tissue disease: Part II: Cardiac, surgical, perioperative, operative, and post-operative challenges and management statements	Bermudez CA, Crespo MM, Shlobin OA, et al. ISHLT consensus document on lung transplantation in patients with connective tissue disease: Part II: Cardiac, surgical, perioperative, operative, and post-operative challenges and management statements. J Heart Lung Transplant. 2021;40(11):1267-1278. doi:10.1016/j.healun.2021.07.016
	2020	Donor Heart and Lung Procurement: A Consensus Statement	Copeland H, Hayanga JWA, Neyrinck A, et al. Donor heart and lung procurement: A consensus statement [published correction appears in J Heart Lung Transplant. 2020 Jul;39(7):734. doi: 10.1016/j.healun.2020.06.001]. J Heart Lung Transplant. 2020;39(6):501-517. doi:10.1016/j.healun.2020.03.020
	2020	Utilization of Hepatitis C Virus–Infected Organ Donors in Cardiothoracic Transplantation	Aslam S, Grossi P, Schlendorf KH, et al. Utilization of hepatitis C virus-infected organ donors in cardiothoracic transplantation: An ISHLT expert consensus statement. J Heart Lung Transplant. 2020;39(5):418-432. doi:10.1016/j.healun.2020.03.004
	2017	Report of the ISHLT Working Group on Primary Lung Graft Dysfunction, part I: Definition and grading-A 2016 Consensus Group statement of the	Snell GI, Yusen RD, Weill D, et al. Report of the ISHLT Working Group on Primary Lung Graft Dysfunction, part I: Definition and grading-A 2016 Consensus Group statement of the International Society for Heart and Lung Transplantation. J Heart Lung

		International Society for Heart and Lung Transplantation	Transplant. 2017;36(10):1097-1103. doi:10.1016/j.healun.2017.07.021
	2017	Report of the International Society for Heart and Lung Transplantation Working Group on Primary Lung Graft Dysfunction, part II: Epidemiology, risk factors, and outcomes-A 2016 Consensus Group statement of the International Society for Heart and Lung Transplantation	Diamond JM, Arcasoy S, Kennedy CC, et al. Report of the International Society for Heart and Lung Transplantation Working Group on Primary Lung Graft Dysfunction, part II: Epidemiology, risk factors, and outcomes-A 2016 Consensus Group statement of the International Society for Heart and Lung Transplantation. J Heart Lung Transplant. 2017;36(10):1104-1113. doi:10.1016/j.healun.2017.07.020
	2017	Report of the ISHLT Working Group on Primary Lung Graft Dysfunction Part III: Mechanisms: A 2016 Consensus Group Statement of the International Society for Heart and Lung Transplantation	Gelman AE, Fisher AJ, Huang HJ, et al. Report of the ISHLT Working Group on Primary Lung Graft Dysfunction Part III: Mechanisms: A 2016 Consensus Group Statement of the International Society for Heart and Lung Transplantation. J Heart Lung Transplant. 2017;36(10):1114-1120. doi:10.1016/j.healun.2017.07.014
	2017	Report of the ISHLT Working Group on primary lung graft dysfunction Part IV: Prevention and treatment: A 2016 Consensus Group statement of the International Society for Heart and Lung Transplantation	Van Raemdonck D, Hartwig MG, Hertz MI, et al. Report of the ISHLT Working Group on primary lung graft dysfunction Part IV: Prevention and treatment: A 2016 Consensus Group statement of the International Society for Heart and Lung Transplantation. J Heart Lung Transplant. 2017;36(10):1121-1136. doi:10.1016/j.healun.2017.07.013
	2013	An official American Thoracic Society/International Society for Heart and Lung Transplantation/Society of Critical Care Medicine/Association of Organ and Procurement Organizations/United Network of Organ Sharing Statement: ethical and policy considerations in organ donation after circulatory determination of death	Gries CJ, White DB, Truog RD, et al. An official American Thoracic Society/International Society for Heart and Lung Transplantation/Society of Critical Care Medicine/Association of Organ and Procurement Organizations/United Network of Organ Sharing Statement: ethical and policy considerations in organ donation after circulatory determination of death. Am J Respir Crit Care Med. 2013;188(1):103-109. doi:10.1164/rccm.201304-0714ST
	2005	Report of the ISHLT Working Group on Primary Lung Graft Dysfunction part I: introduction and methods	Christie JD, Van Raemdonck D, de Perrot M, et al. Report of the ISHLT Working Group on Primary Lung Graft Dysfunction part I: introduction and methods. J Heart Lung Transplant. 2005;24(10):1451-1453. doi:10.1016/j.healun.2005.03.004
	2005	Report of the ISHLT Working Group on Primary Lung Graft Dysfunction part II: definition. A consensus statement of the International Society for Heart and Lung Transplantation	Christie JD, Carby M, Bag R, et al. Report of the ISHLT Working Group on Primary Lung Graft Dysfunction part II: definition. A consensus statement of the International Society for Heart and Lung Transplantation. J Heart Lung Transplant. 2005;24(10):1454-1459. doi:10.1016/j.healun.2004.11.049

	2005	Report of the ISHLT Working Group on Primary Lung Graft Dysfunction part III: donor-related risk factors and markers	de Perrot M, Bonser RS, Dark J, et al. Report of the ISHLT Working Group on Primary Lung Graft Dysfunction part III: donor-related risk factors and markers. J Heart Lung Transplant. 2005;24(10):1460-1467. doi:10.1016/j.healun.2005.02.017
	2005	Report of the ISHLT Working Group on Primary Lung Graft Dysfunction part IV: recipient-related risk factors and markers	Barr ML, Kawut SM, Whelan TP, et al. Report of the ISHLT Working Group on Primary Lung Graft Dysfunction part IV: recipient-related risk factors and markers. J Heart Lung Transplant. 2005;24(10):1468-1482. doi:10.1016/j.healun.2005.02.019
	2005	Report of the ISHLT Working Group on Primary Lung Graft Dysfunction part V: predictors and outcomes	Arcasoy SM, Fisher A, Hachem RR, Scavuzzo M, Ware LB; ISHLT Working Group on Primary Lung Graft Dysfunction. Report of the ISHLT Working Group on Primary Lung Graft Dysfunction part V: predictors and outcomes. J Heart Lung Transplant. 2005;24(10):1483-1488. doi:10.1016/j.healun.2004.11.314
	2005	Report of the ISHLT Working Group on Primary Lung Graft Dysfunction part VI: treatment	Shargall Y, Guenther G, Ahya VN, et al. Report of the ISHLT Working Group on Primary Lung Graft Dysfunction part VI: treatment. J Heart Lung Transplant. 2005;24(10):1489-1500. doi:10.1016/j.healun.2005.03.011
	2003	A Review of Lung Transplant Donor Acceptability Criteria	Orens JB, Boehler A, de Perrot M, et al. A review of lung transplant donor acceptability criteria. J Heart Lung Transplant. 2003;22(11):1183-1200. doi:10.1016/s1053-2498(03)00096-2
**Posttransplant**	2022	Reproductive health after thoracic transplantation: An ISHLT expert consensus statement	Kittleson MM, DeFilippis EM, Bhagra CJ, et al. Reproductive health after thoracic transplantation: An ISHLT expert consensus statement. J Heart Lung Transplant. 2023;42(3):e1-e42. doi:10.1016/j.healun.2022.10.009
	2022	Consensus recommendations for use of maintenance immunosuppression in solid organ transplantation: Endorsed by the American College of Clinical Pharmacy, American Society of Transplantation, and the International Society for Heart and Lung Transplantation	Nelson J, Alvey N, Bowman L, et al. Consensus recommendations for use of maintenance immunosuppression in solid organ transplantation: Endorsed by the American College of Clinical Pharmacy, American Society of Transplantation, and the International Society for Heart and Lung Transplantation. Pharmacotherapy. 2022;42(8):599-633. doi:10.1002/phar.2716
	2021	Cystic fibrosis foundation consensus statements for the care of cystic fibrosis lung transplant recipients	Shah P, Lowery E, Chaparro C, et al. Cystic fibrosis foundation consensus statements for the care of cystic fibrosis lung transplant recipients. J Heart Lung Transplant. 2021;40(7):539-556. doi:10.1016/j.healun.2021.04.011
	2021	ISHLT consensus document on lung transplantation in patients with	Crespo MM, Claridge T, Domsic RT, et al. ISHLT consensus document on lung transplantation in

		connective tissue disease: Part III: Pharmacology, medical and surgical management of post-transplant extrapulmonary conditions statements	patients with connective tissue disease: Part III: Pharmacology, medical and surgical management of post-transplant extrapulmonary conditions statements. J Heart Lung Transplant. 2021;40(11):1279-1300. doi:10.1016/j.healun.2021.07.013
	2020	ISHLT Consensus Statement for the Standardization of Bronchoalveolar Lavage in Lung Transplantation	Martinu T, Koutsokera A, Benden C, et al. International Society for Heart and Lung Transplantation consensus statement for the standardization of bronchoalveolar lavage in lung transplantation. J Heart Lung Transplant. 2020;39(11):1171-1190. doi:10.1016/j.healun.2020.07.006
	2020	Initial skin cancer screening for solid organ transplant recipients in the United States: Delphi method development of expert consensus guidelines	Crow LD, Jambusaria-Pahlajani A, Chung CL, et al. Initial skin cancer screening for solid organ transplant recipients in the United States: Delphi method development of expert consensus guidelines. Transpl Int. 2019;32(12):1268-1276. doi:10.1111/tri.13520
	2019	Chronic lung allograft dysfunction: Definition and update of restrictive allograft syndrome-A consensus report from the Pulmonary Council of the ISHLT	Glanville AR, Verleden GM, Todd JL, et al. Chronic lung allograft dysfunction: Definition and update of restrictive allograft syndrome-A consensus report from the Pulmonary Council of the ISHLT. J Heart Lung Transplant. 2019;38(5):483-492. doi:10.1016/j.healun.2019.03.008
	2019	Chronic lung allograft dysfunction: Definition, diagnostic criteria, and approaches to treatment-A consensus report from the Pulmonary Council of the ISHLT	Verleden GM, Glanville AR, Lease ED, et al. Chronic lung allograft dysfunction: Definition, diagnostic criteria, and approaches to treatment-A consensus report from the Pulmonary Council of the ISHLT. J Heart Lung Transplant. 2019;38(5):493-503. doi:10.1016/j.healun.2019.03.009
	2018	ISHLT Consensus Statement on Adult and Pediatric Airway Complications after Lung Transplantation	Crespo MM, McCarthy DP, Hopkins PM, et al. ISHLT Consensus Statement on adult and pediatric airway complications after lung transplantation: Definitions, grading system, and therapeutics. J Heart Lung Transplant. 2018;37(5):548-563. doi:10.1016/j.healun.2018.01.1309
	2016	The 2015 International Society for Heart and Lung Transplantation Guidelines for the management of fungal infections in mechanical circulatory support and cardiothoracic organ transplant recipients: Executive summary	Husain S, Sole A, Alexander BD, et al. The 2015 International Society for Heart and Lung Transplantation Guidelines for the management of fungal infections in mechanical circulatory support and cardiothoracic organ transplant recipients: Executive summary. J Heart Lung Transplant. 2016;35(3):261-282. doi:10.1016/j.healun.2016.01.007
	2016	Antibody-mediated rejection of the lung: A consensus report of the International Society for Heart and Lung Transplantation	Levine DJ, Glanville AR, Aboyoun C, et al. Antibody-mediated rejection of the lung: A consensus report of the International Society for Heart and Lung Transplantation. J Heart Lung Transplant. 2016;35(4):397-406.

			doi:10.1016/j.healun.2016.01.1223
	2015	Adult cardiothoracic transplant nursing: an ISHLT consensus document on the current adult nursing practice in heart and lung transplantation	Coleman B, Blumenthal N, Currey J, et al. Adult cardiothoracic transplant nursing: an ISHLT consensus document on the current adult nursing practice in heart and lung transplantation. J Heart Lung Transplant. 2015;34(2):139-148. doi:10.1016/j.healun.2014.11.017
	2014	An international ISHLT/ATS/ERS clinical practice guideline: diagnosis and management of bronchiolitis obliterans syndrome	Meyer KC, Raghu G, Verleden GM, et al. An international ISHLT/ATS/ERS clinical practice guideline: diagnosis and management of bronchiolitis obliterans syndrome. Eur Respir J. 2014;44(6):1479-1503. doi:10.1183/09031936.00107514
	2010	A 2010 Working Formulation for the Standardization of Definitions of Infections in Cardiothoracic Transplant Recipients	Husain S, Mooney ML, Danziger-Isakov L, et al. A 2010 working formulation for the standardization of definitions of infections in cardiothoracic transplant recipients. J Heart Lung Transplant. 2011;30(4):361-374. doi:10.1016/j.healun.2011.01.701
	2009	Generic drug immunosuppression in thoracic transplantation: an ISHLT educational advisory	Uber PA, Ross HJ, Zuckermann AO, et al. Generic drug immunosuppression in thoracic transplantation: an ISHLT educational advisory. J Heart Lung Transplant. 2009;28(7):655-660. doi:10.1016/j.healun.2009.05.001
	2007	Revision of the 1996 working formulation for the standardization of nomenclature in the diagnosis of lung rejection	Stewart S, Fishbein MC, Snell GI, et al. Revision of the 1996 working formulation for the standardization of nomenclature in the diagnosis of lung rejection. J Heart Lung Transplant. 2007;26(12):1229-1242. doi:10.1016/j.healun.2007.10.017
	2006	Present Status of Research on Psychosocial Outcomes in Cardiothoracic Transplantation—Review and Recommendations for the Field	Psychosocial Outcomes Workgroup of the Nursing and Social Sciences Council of the International Society for Heart and Lung Transplantation, Cupples S, Dew MA, et al. Report of the Psychosocial Outcomes Workgroup of the Nursing and Social Sciences Council of the International Society for Heart and Lung Transplantation: present status of research on psychosocial outcomes in cardiothoracic transplantation: review and recommendations for the field. J Heart Lung Transplant. 2006;25(6):716-725. doi:10.1016/j.healun.2006.02.005
	2001	Bronchiolitis Obliterans Syndrome 2001: An Update of the Diagnostic Criteria	Estenne M, Maurer JR, Boehler A, et al. Bronchiolitis obliterans syndrome 2001: an update of the diagnostic criteria. J Heart Lung Transplant. 2002;21(3):297-310. doi:10.1016/s1053-2498(02)00398-4
	2000	Report of the Xenotransplantation Advisory Committee of the	Cooper DK, Keogh AM, Brink J, et al. Report of the Xenotransplantation Advisory Committee of the International Society for Heart and Lung

		International Society for Heart and Lung Transplantation: the present status of xenotransplantation and its potential role in the treatment of end-stage cardiac and pulmonary diseases	Transplantation: the present status of xenotransplantation and its potential role in the treatment of end-stage cardiac and pulmonary diseases. J Heart Lung Transplant. 2000;19(12):1125-1165. doi:10.1016/s1053-2498(00)00224-2
	1998	ISHLT/ATS/ASTP/ERS International Guidelines for the Selection of Lung Transplant Candidates	International guidelines for the selection of lung transplant candidates. The American Society for Transplant Physicians (ASTP)/American Thoracic Society(ATS)/European Respiratory Society(ERS)/International Society for Heart and Lung Transplantation(ISHLT). Am J Respir Crit Care Med. 1998;158(1):335-339. doi:10.1164/ajrccm.158.1.15812

Supplementary Table 2: Diagnoses included within each of the 4 main indication groups.

Indication group	Included diagnoses
**Obstructive**	chronic obstructive pulmonary disease, emphysema, bronchiolitis obliterans, non-cystic bronchiectasis, bronchiolitis obliterans syndrome, lymphangioleiomyomatosis
**Restrictive**	interstitial lung diseases, anthracosilicosis, restrictive allograft syndrome, mixed phenotype of chronic lung allograft syndrome, acute respiratory distress syndrome
**Vascular**	congenital heart malformations, Eisenmenger's syndrome, idiopathic pulmonary hypertension, chronic thromboembolic pulmonary disease, pulmonary veno-occlusive disease, hemangioendothelioma, stenosis of arteria pulmonalis
**Cystic fibrosis**	cystic fibrosis

Supplementary Table 3: Demographic data of patients who underwent lung transplantation (LuTx), grouped based on time periods.

	ALL (1991-2024)	1991-2000	2001-2010	2011-2020	2021-2024	p
**Number of LuTx, n (%)**	1,500 (100)	118 (8)	450 (30)	670 (45)	262 (17)	-
**Type of procedure**						**<0.0001**
Unilateral, n (%)	160 (11)	53 (45)	97 (21)	3 (1)	7 (3)	
Bilateral, n (%)	1,267 (84)	40 (34)	332 (74)	644 (96)	251 (96)	
Combined, n (%)	73 (5)	25 (21)	21 (5)	23 (3)	4 (2)	
**Female sex, n (%)**	712 (48)	51 (43)	214 (48)	333 (50)	114 (44)	0.74
**Age at LuTx in years, median (IQR)**	56 (45 – 61)	48 (39 -55)	54 (40 - 59)	58 (47 - 61)	60 (54 - 63)	**<0.0001**
**Type of donation**						**<0.0001**
DBD, n (%)	1,213 (81)	118 (100)	425 (94)	513 (77)	157 (60)	
DCD, n (%)	287 (19)	0	25 (6)	157 (23)	105 (40)	
**Age of donor in years, median (IQR)**	48 (35 – 58)	33 (21 - 42)	44 (29 - 52)	52 (39 - 61)	56 (44 - 67)	**<0.0001**
**Indication diagnosis**						**<0.0001**
Obstructive, n (%)	852 (57)	57 (48)	249 (55)	407 (61)	139 (53)	
Restrictive, n (%)	362 (24)	22 (19)	99 (22)	140 (21)	101 (39)	
Vascular, n (%)	106 (7)	27 (23)	38 (8)	29 (4)	12 (5)	

Cystic fibrosis, n (%)	180 (12)	12 (10)	64 (14)	94 (14)	10 (4)	
**Graft survival**						
30-days, n (%)	1451 (97)	107 (91)	436 (97)	658 (98)	250 (98)	**0.005**
1-year, n (%)	1254 (88)	84 (71)	402 (89)	606 (90)	162 (87)	**<0.0001**
3-year, n (%)	1006 (78)	62 (53)	347 (77)	553 (83)	44 (81)	**<0.0001**
5-year, n (%)	817 (70)	53 (45)	318 (71)	446 (74)	-	**<0.0001**

Supplementary Table 4: Demographic data of patients who underwent lung retransplantation (reLuTx), grouped based on time periods.

	**All (1991-2024)**	**1991-2000**	**2001-2010**	**2011-2020**	**2021-2024**	**p**
**Number of reLuTx, n (%)**	59 (100)	2 (4)	18 (31)	34 (58)	5 (5)	-
**Type of procedure**						**<0.01**
Unilateral, n (%)	6 (10)	2 (100)	2 (11)	2 (6)	0	
Bilateral, n (%)	52 88)	0	16 (89)	31 (91)	5 (100)	
Combined, n (%)	1 (2)	0	0	1 (3)	0	
**Type of donation**						0.93
DBD, n (%)	47 (80)	2 (100)	15 (83)	26 (76)	4 (80)	
DCD, n (%)	12 (20)	0	3 (17)	8 (24)	1 (20)	
**Age of donor in years, median (IQR)**	49 (39 – 56)	47 (N/A)	46 (35 - 52)	52 (40 - 58)	53 (30- 58)	0.41
**Female sex, n (%)**	32 (54)	2 (100)	13 (72)	14 (42)	3 (60)	0.30
**Age at reLuTx in years, median (IQR)**	42 (30 – 53)	48 (N/A)	36 (23 -54)	44 (31 - 52)	43 (31 - 52)	0.51
**Indication diagnosis**						0.38
Obstructive, n (%)	45 (76)	2 (100)	17 (94)	23 (68)	3 (60)	
Restrictive, n (%)	13 (22)	0	1 (6)	10 (29)	2 (40)	
Vascular, n (%)	1 (2)	0	0	1 (3)	0	

**Graft survival**						
30-days, n (%)	57 (97)	1 (50)	18 (100)	33 (97)	5 (100)	**0.003**
1-year, n (%)	47 (83)	1 (50)	16 (89)	28 (83)	2 (75)	0.07
3-year, n (%)	39 (72)	1 (50)	12 (67)	26 (77)	-	0.009
5-year, n (%)	31 (57)	1 (50)	10 (56)	20 (59)	-	0.11

Supplementary Table 5: Practical guidelines and consensus statements currently in development on pre, peri-, and post-transplant care of lung transplant recipients, endorsed by the International Society for Heart and Lung Transplantation.

**Pretransplant care**
Assessing and Addressing Frailty in Candidates for Lung Transplantation: A Consensus Statement
ISHLT Consensus Document for the Referral and Selection of Pediatric Lung Transplant Candidates
Consensus Statement on Clinical Use of Risk Stratification Tools in the Management of Pulmonary Arterial Hypertension
Consensus Document on Xenotransplantation in the Treatment of End-Stage Cardiac and Pulmonary Disease
**Peritransplant care**
Engagement of Social Support Systems in Patients being Considered for and Following Advanced Heart and Lung Therapies: An ISHLT Expert Consensus Statement
ISHLT Consensus Statement on the Perioperative use of ECLS in Lung Transplantation: Part I - Preoperative Considerations
Guidelines for Processing Explanted Native and Allograft Lungs
**Posttransplant care**
ISHLT Consensus Statement on Acute Lung Allograft Dysfunction: Definition, Diagnostic and Therapeutic Approaches, and Areas for Research
Baseline Lung Allograft Dysfunction: Definition, Timing, and Implications for Outcomes - An ISHLT Consensus Document
Diagnosis and Management of Short Telomere Syndrome in Lung Transplantation
Antibody-Mediated Rejection after Lung Transplantation: A Consensus Report of the International Society for Heart and Lung Transplantation
ISHLT 2025 Working Classification of Lung Allograft Pathology
ISHLT Statement on Surrogate Endpoints in Lung Transplant Trials
Palliative Care in Patients Prior to or Following Heart Transplantation, Lung Transplantation, or Mechanical Circulatory Support Implantation: An ISHLT Expert Consensus Statement
